# Highly Pathogenic Avian Influenza A(H5N8) Clade 2.3.4.4b Virus in Dust Samples from Poultry Farms, France, 2021

**DOI:** 10.3201/eid2807.212247

**Published:** 2022-07

**Authors:** Fabien Filaire, Laetitia Lebre, Charlotte Foret-Lucas, Timothée Vergne, Patrick Daniel, Aurélie Lelièvre, Antoine de Barros, Adam Jbenyeni, Pierrick Bolon, Mathilde Paul, Guillaume Croville, Jean-Luc Guérin

**Affiliations:** THESEO France, Laval, France (F. Filaire);; Université de Toulouse, Toulouse, France (F. Filaire, L. Lebre, C. Foret-Lucas, T. Vergne, A. De Barros, A. Jbenyeni, P. Bolon, M. Paul, G. Croville, J.-L. Guérin);; Laboratoires des Pyrénées et des Landes, Mont-de-Marsan, France (P. Daniel);; SOCSA 40, Amou, France (A. Lelièvre)

**Keywords:** Influenza, viruses, zoonoses, respiratory infections, highly pathogenic avian influenza virus, H5N8, aerosol, dust, environment, surveillance, poultry, France

## Abstract

Avian influenza A(H5N8) virus has caused major epizootics in Europe since 2016. We conducted virologic analysis of aerosol and dust collected on poultry farms in France during 2020–2021. Our results suggest dust contributes to viral dispersal, even early in an outbreak, and could be a valuable surveillance tool.

Avian influenza is a viral disease caused by influenza A viruses, segmented, negative, single-stranded RNA viruses belonging to the *Orthomyxoviridae* family. Wild aquatic birds are the virus reservoir and generate occasional worldwide panzootic outbreaks during seasonal migrations (*1*). Highly pathogenic avian influenza (HPAI) virus subtypes can cause panzootic outbreaks associated with high mortality in wild and domestic birds, as well as substantial economic losses for the poultry industry, and are a major threat to public health because of their zoonotic potential.

During winter 2020–21, the HPAI H5N8 virus belonging to the A/goose/Guangdong/1/1996 clade 2.3.4.4b lineage caused hundreds of outbreaks among wild and domestic flocks across Europe (*2*,*3*). France was severely affected; 492 poultry farms, primarily duck farms, were infected during December 5, 2020–May 3, 2021. Despite reinforced surveillance activities, the virus spread rapidly, posing major challenges for surveillance and control. Officially recognized surveillance methods involve tracheal or cloacal swab-based sampling (*4*,*5*). However, these methods are laborious and have technical requirements that make application on such a massive scale difficult; thus, newer surveillance methods are needed. 

Epidemiologic modeling of this outbreak suggested within-farm viral transmission was extremely fast, and the environment was a major source of contamination for neighboring farms (*6*). HPAI viruses disperse in aerosols, in fomites carried by human and animal vectors, and via feathers, fecal particles, and to a great extent, dust (*7*–*9*). Poultry farms are known to heavily generate dust particles that spread from feed, litter, feces, and animal skin and feathers (*9*,*10*). These particles can act as vehicles for bacteria and viruses and are classified, depending on their size, as inhalable (<100 µm), thoracic (<10 µm), or respirable (<4 µm) (*10*). In poultry houses, most dust consists of nonrespirable particles >4 µm (*10*). We evaluated the role of dust as a vehicle of H5N8 clade 2.3.4.4b virus and assessed whether dust or aerosol sampling is a viable alternative to bird swab sampling for HPAI virus surveillance.

## The Study

During December 2020–April 2021, we conducted a study in 63 poultry houses located in 4 departments (administrative units) in France highly affected by HPAI H5N8 virus outbreaks. On the basis of daily official outbreak reports, we identified HPAI-infected poultry houses and poultry houses in close vicinity or with epidemiologic links to infected houses. The study included a total of 48 duck houses, 12 chicken houses, 2 quail houses, and 1 goose house. We selected farms identified as being near an HPAI outbreak to reflect a range of sanitary statuses and infection stages (i.e., no, mild, or severe clinical signs; high mortality rates). We specifically included houses without clinical signs among animals to evaluate virus dispersal and dust testing for HPAI surveillance in the early stages of infection.

In each selected poultry house, we collected surface dust with 2 wipes on the building’s walls and feeders (*9*,*11*) (Appendix). In 19 houses, we also collected aerosol samples by using 2 devices, Coriolis Compact (Bertin Instruments, https://www.bertin-instruments.com) and the NIOSH BC 251 developed by the National Institute for Occupational Safety and Health (NIOSH; https://www.cdc.gov/niosh) (Appendix). Furthermore, we collected tracheal swab samples from 20 randomly selected birds in each house (Appendix Table 1). We chose tracheal over cloacal swab samples because the typical respiratory shedding and tropism of HPAI H5N8 clade 2.3.4.4 viruses enables earlier detection in the respiratory tract than cloacae (*12*,*13*).

**Figure 1:**
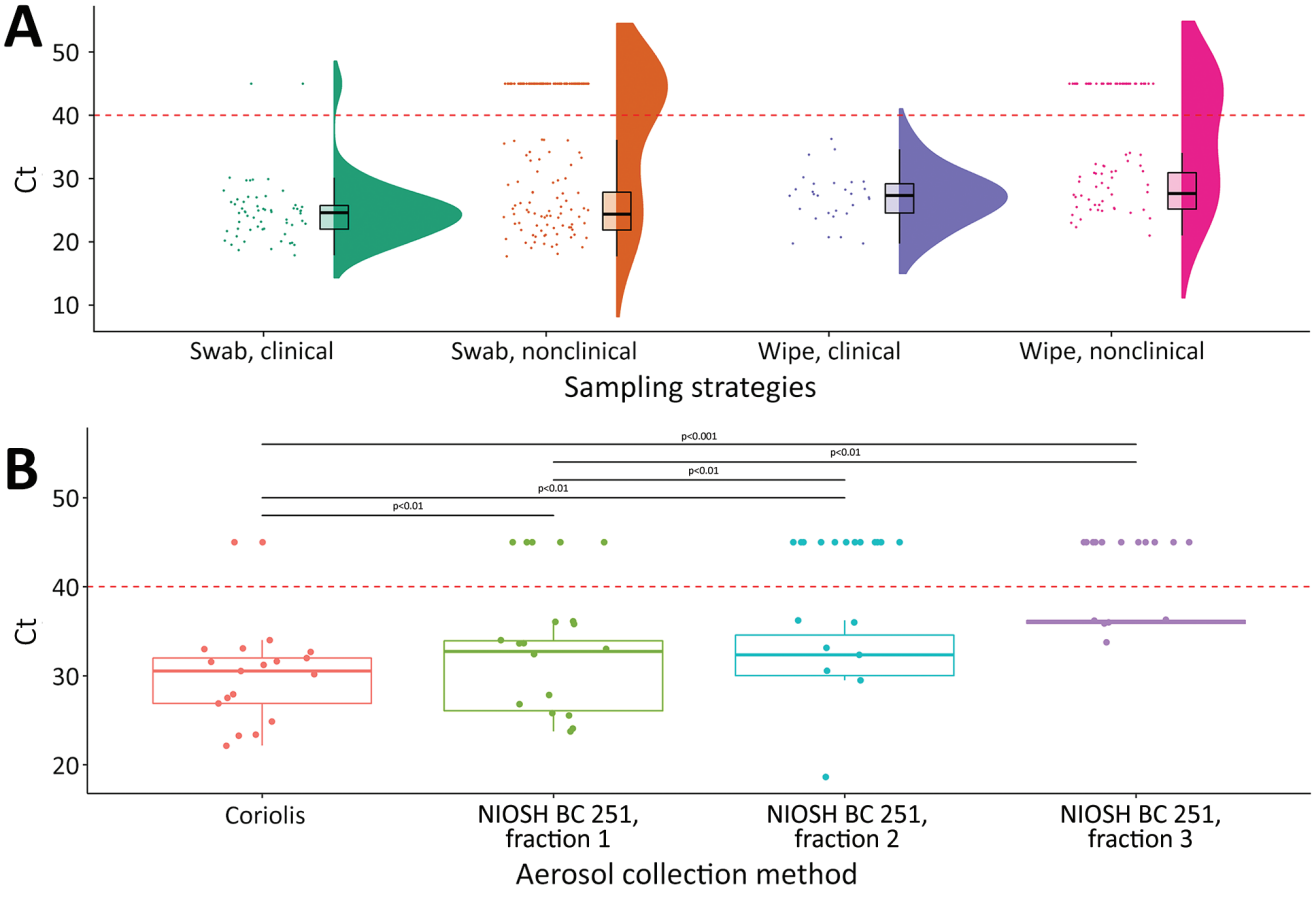
Ct values of highly pathogenic avian influenza A(H5N8) clade 2.3.4.4b virus detected by real-time qualitative reverse transcription PCR from tracheal swab and environmental samples collected on poultry farms, France, December 2020–April 2021. We used a Wilcoxon test for statistical analysis and considered samples with Ct <40 negative. Each dot indicates a Ct value from 1 wipe sample or 1 pool of 5 tracheal swab samples. Box plots show 95% CI for Ct values; horizontal lines in boxes indicate means and error bars SDs. Red dashed horizontal lines indicate Ct of 40, the cutoff value for negative results. A) Half-violin, scatter, and box plots of Ct values for samples collected by using tracheal swab samples or surface wipe samples from 63 poultry houses with and without clinical signs among animals. Half-violins show distribution of Ct values for each sample type. B) C_t_ values for aerosol samples collected in 19 poultry houses. Aerosol samples were collected by using the Coriolis Compact (Bertin Instruments, https://www.bertin-instruments.com) and the NIOSH BC 251 (https://www.cdc.gov/niosh). The NIOSH BC 251 sampling device has 3 fractions for different particle sizes; fraction 1 for >4 µm, fraction 2 for 1–4 µm, and fraction 3 for <1 µm. Ct, cycle threshold; NIOSH, National Institute for Occupational Safety and Health.

We performed real-time quantitative reverse transcription PCR on all samples to detect HPAI virus at the molecular level by targeting the matrix protein and H5 genes (Appendix). We compared cycle threshold (Ct) distributions of each sample by using raincloud plots and a boxplot model (Figure 1). In general, Ct values for tracheal swabs (≈25.2) and dust (≈28.6) were similar (Figure 1; Appendix). Between the 2 aerosol collectors, the Coriolis device showed more positive results (Ct <40) than the NIOSH BC 251 sampler. Furthermore, we noted HPAI H5N8 virus was more easily detected in the largest particles, those >1 µm (Figure 1). These results suggest that the HPAI H5N8 virus dispersion is associated with large dust particles, which could be a major vehicle for viral spread.

**Figure 2:**
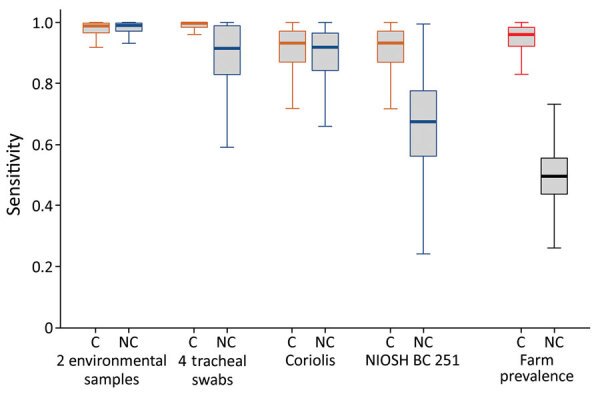
Sensitivity comparison of 4 sampling techniques used to detect highly pathogenic avian influenza A(H5N8) clade 2.3.4.4b virus from 63 poultry farms, France, December 2020–April 2021. Sampling was conducted in poultry houses with and without clinical signs among flocks. Box plots show 95% CIs; horizontal lines in boxes indicate means, error bars SDs. The 2 environmental samples refer to 2 wipes collected in the animal houses, 1 on feeders and 1 on walls. Tracheal swab samples refer to 4 pools of 5 swab samples collected per house. Aerosol samples were collected from 19 poultry houses by using the Coriolis Compact (Bertin Instruments, https://www.bertin-instruments.com) and the NIOSH BC 251 (https://www.cdc.gov/niosh). The NIOSH BC 251 sampling device has 3 fractions for different particle sizes; fraction 1 for >4 µm, fraction 2 for 1–4 µm, and fraction 3 for <1 µm. Farm-level disease prevalence was 0.96 for houses in which animals had clinical signs and 0.5 in houses in which animals did not have clinical signs. C, clinical signs; NC, no clinical signs; NIOSH, National Institute for Occupational Safety and Health.

To estimate the sensitivity of the 4 different sampling methods (tracheal swab samples, surface wipes, and Coriolis and NIOSH aerosol samplers) in houses with or without poultry showing clinical signs, we used a latent class modeling approach, necessary when no standard has been established (*14*). We adjusted the model to cross-detect each farm by the 4 different sampling methods and estimated model parameters in a Bayesian framework (Appendix). Model outputs suggested that the different sampling methods had equivalent sensitivity in HPAI-infected flocks showing clinical signs. Surface dust and aerosol sampling showed substantially higher sensitivity in HPAI-infected flocks without clinical signs, but the difference was not statistically significant despite overlap of 95% credible intervals (Table 1; Figure 2).

**Table 1:** Estimated sensitivity of sampling methods and sampling strategies by latent class analysis for detection of highly pathogenic avian influenza A(H5N8) virus on poultry farms, France, December 2020–April 2021

Clinical signs	Samples*	Estimated sensitivity of sampling method (95% credible interval)†	Estimated sensitivity of sampling strategy (95% credible interval)‡
Clinical signs in flock	Tracheal swab	0.77 (0.44–0.99)	1.00 (0.90–1.00)
	Wipe	0.89 (0.64–1.00)	0.99 (0.87–1.00)
	Coriolis	0.93 (0.69–1.00)	0.93 (0.69–1.00)
	NIOSH BC 251	0.93 (0.69–1.00)	0.93 (0.69–1.00)
No clinical signs in flock	Tracheal swab	0.46 (0.15–0.97)	0.92 (0.48–1.00)
	Wipe	0.90 (0.67–1.00)	0.99 (0.89–1.00)
	Coriolis	0.92 (0.63–1.00)	0.92 (0.63–1.00)
	NIOSH BC 251	0.67 (0.34–0.91)	0.67(0.34–0.91)

*Each farm or building was sampled by using 20 tracheal swab samples (pooled in sets of 5 for RT-PCR) and 2 wipe samples from surfaces; on 19 farms we also collected 1 air sample from each of the 2 aerosol collection devices, the Coriolis Compact (Bertin Instruments, https://www.bertin-instruments.com) and the NIOSH BC 251 developed by the National Institute for Occupational Safety and Health (https://www.cdc.gov/niosh). †Sampling method relates to the simple analysis of individual samples; individual tracheal swab samples are those analyzed in pools of 5 samples; thus, the sensitivity of the sampling method corresponds to the probability that a single sample, or a pooled sample for the tracheal swabs, tests positive in an infected poultry house. ‡Sampling strategy relates to the combined analysis of the different individual samples at the farm or building level, assuming that the farm or building is positive when >1 individual sample tests positive; thus, the sensitivity of the sampling strategy corresponds to the probability that >1 sample, or >1 pool of 5 tracheal swab samples, tests positive in an infected poultry house.

Finally, to assess the infectiousness of environmental samples, we processed 25 surface dust or aerosol samples taken from 5 animal houses and used these for virus isolation in embryonated eggs (Appendix). Among 25 samples, 12 (48%) tested positive, confirming that viral isolation is possible from these sampling methods (Table 2).

**Table 2:** Viral isolation assays on chicken embryonated eggs performed on 5 of the 63 poultry houses in a study to detect highly pathogenic avian influenza A(H5N8) virus on poultry farms, France, December 2020–April 2021*

Sample type†	House 11			House 26			House 29			House 30			House 34	
	Ct	VI		Ct	VI		Ct	VI		Ct	VI		Ct	VI
Tracheal swab	25	+		20.7	+		21.9	+		18.9	+		20	+
Dust wipe, feeders	25.8	–		25.1	–		27.4	+		29.5	+		24.2	+
Dust wipe, walls	27.5	+		25.5	–		30.1	+		28.3	+		23	+
Coriolis	32	–		33.6	–		27.8	–		25.8	+		26.9	+
NIOSH BC251														
Fraction 1	34	–		33.6	–		27.8	–		25.8	+		23.7	+
Fraction 2	–	ND		36	–		32.4	–		33.1	–		18.6	+
Fraction 3	–	ND		–	ND		36.3	–		–	ND		–	ND

*Ct, cycle threshold; ND, not done; VI, virus isolation; +, positive; –, negative. †Each farm or building was sampled by using 4 pools of 5 tracheal swab samples, 2 wipe samples (1 from feeders, 1 from walls), and on 19 farms, 1 air sample from each of the 2 aerosol collection devices, the Coriolis Compact (Bertin Instruments, https://www.bertin-instruments.com) and the NIOSH BC 251, developed by the National Institute for Occupational Safety and Health (https://www.cdc.gov/niosh). NIOSH BC 251 sampling device has 3 fractions for different particle sizes; fraction 1 for >4 µm, fraction 2 for 1–4 µm, and fraction 3 for <1 µm.

## Conclusions

We used field conditions to evaluate whether dust from poultry farms contained HPAI viruses and to compare surface dust and aerosol testing for HPAI virus against official swab-based methods. We used wipe tests to collect surface dust and 2 bioaerosol devices to collect aerosol samples during the 2020–21 HPAI H5N8 virus epizootic outbreak in France. Standard molecular analysis detected high viral RNA loads in the early phase of flock infection, before clinical signs appeared. In addition, size fractioning of aerosol samples revealed that high RNA viral loads and infectious viral particles were associated with the largest particles (>1 µm), which are easy to collect and use for molecular analysis. However, the field conditions we used cannot be reproduced in experimental animal trials because of ethical and biosecurity requirements, which result in dramatically lower dust loads than those found in the field.

Recent research on influenza transmission routes revealed that nonrespiratory airborne particles are more likely to cause infection than are droplets or fomites (*7*). Infectious aerosols generated from inert objects handled by humans or dispersed through animal movements can lead to further infection. Dust can carry infectious particles and is omnipresent in poultry houses (*10*) and so could be a major means of viral transmission and dispersal in the environment. These findings suggest that biosecurity protocols should strongly emphasize limiting the amount of dust dispersed via farm equipment to reduce the spread of HPAI viruses.

Of note, for early detection, before flock animals show clinical signs of illness, we found that surface dust sampling using wipe tests and aerosol sampling using a high flow rate collection device are more sensitive than tracheal swab samples. The higher sensitivity of environmental sampling methods for early detection is likely because of infection dynamics at the flock level. During the early phases of infection, only a few animals are infectious, making the probability of detecting virus during individual swab-based sampling low (*6*). Swab sampling also is time consuming, labor-intensive, and expensive, whereas dust wiping is inexpensive, fast, easy to perform, and noninvasive. 

In conclusion, we detected HPAI H5N8 clade 2.3.4.4b virus in dust samples from poultry farms during a large epizootic in France. Our findings suggest dust wipe samples are an efficient surveillance tool and could enable more rapid virus detection and implementation of measures to curb virus spread.

Appendix Additional information on detection of highly pathogenic avian influenza A(H5N8) clade 2.3.4.4b virus in dust samples from poultry farms, France, 2021.

## References

[1] Munster VJ, Baas C, Lexmond P, Waldenström J, Wallensten A, Fransson T, et al. Spatial, temporal, and species variation in prevalence of influenza A viruses in wild migratory birds. PLoS Pathog. 2007;3:e61. 10.1371/journal.ppat.0030061 17500589 PMC1876497

[2] Lewis NS, Banyard AC, Whittard E, Karibayev T, Al Kafagi T, Chvala I, et al. Emergence and spread of novel H5N8, H5N5 and H5N1 clade 2.3.4.4 highly pathogenic avian influenza in 2020. Emerg Microbes Infect. 2021;10:148–51. 10.1080/22221751.2021.1872355 33400615 PMC7832535

[3] Adlhoch C, Fusaro A, Kuiken T, Niqueux É, Staubach C, Terregino C, et al.; European Food Safety Authority; European Centre for Disease Prevention and Control and European Union Reference Laboratory for Avian Influenza. Avian influenza overview May - August 2020. EFSA J. 2020;18:e06270. 33281980 10.2903/j.efsa.2020.6270 PMC7525800

[4] World Organisation for Animal Health (OIE). Avian influenza (including infection with high pathogenicity avian influenza viruses). In OIE terrestrial manual 2021. Geneva: The Organisation; 2021.

[5] Nielsen SS, Alvarez J, Bicout DJ, Calistri P, Depner K, Drewe JA, et al.; EFSA Panel on Animal Health and Welfare (EFSA AHAW Panel). (EFSA AHAW Panel). Scientific opinion on the assessment of the control measures of the category A diseases of Animal Health Law: Highly Pathogenic Avian Influenza. EFSA J. 2021;19:e06372. 33488812 10.2903/j.efsa.2021.6372 PMC7812451

[6] Vergne T, Gubbins S, Guinat C, Bauzile B, Delpont M, Chakraborty D, et al. Inferring within-flock transmission dynamics of highly pathogenic avian influenza H5N8 virus in France, 2020. Transbound Emerg Dis. 2021;68:3151–5. 10.1111/tbed.14202 34170081 PMC9291964

[7] Asadi S, Gaaloul Ben Hnia N, Barre RS, Wexler AS, Ristenpart WD, Bouvier NM. Influenza A virus is transmissible via aerosolized fomites. Nat Commun. 2020;11:4062. 10.1038/s41467-020-17888-w 32811826 PMC7435178

[8] Spekreijse D, Bouma A, Koch G, Stegeman A. Quantification of dust-borne transmission of highly pathogenic avian influenza virus between chickens. Influenza Other Respir Viruses. 2013;7:132–8. 10.1111/j.1750-2659.2012.00362.x 22487257 PMC4986625

[9] Lopez KM, Nezworski J, Rendahl A, Culhane M, Flores-Figueroa C, Muñoz-Aguayo J, et al. Environmental sampling survey of H5N2 highly pathogenic avian influenza–infected layer chicken farms in Minnesota and Iowa. Avian Dis. 2018;62:373–80. 10.1637/11891-050418-Reg.1 31119921

[10] Zhao Y, Aarnink AJA, De Jong MCM, Groot Koerkamp PWG. Airborne microorganisms from livestock production systems and their relation to dust. Crit Rev Environ Sci Technol. 2014;44:1071–128. 10.1080/10643389.2012.746064 32288664 PMC7113898

[11] Carrique-Mas JJ, Breslin M, Sayers AR, McLaren I, Arnold M, Davies R. Comparison of environmental sampling methods for detecting *Salmonella* in commercial laying flocks in the UK. Lett Appl Microbiol. 2008;47:514–9. 10.1111/j.1472-765X.2008.02450.x 19120919

[12] Gaide N, Foret-Lucas C, Figueroa T, Vergne T, Lucas MN, Robertet L, et al. Viral tropism and detection of clade 2.3.4.4b H5N8 highly pathogenic avian influenza viruses in feathers of ducks and geese. Sci Rep. 2021;11:5928. 10.1038/s41598-021-85109-5 33723295 PMC7960704

[13] Beerens N, Germeraad EA, Venema S, Verheij E, Pritz-Verschuren SBE, Gonzales JL. Comparative pathogenicity and environmental transmission of recent highly pathogenic avian influenza H5 viruses. Emerg Microbes Infect. 2021;10:97–108. 10.1080/22221751.2020.1868274 33350337 PMC7832006

[14] van Smeden M, Naaktgeboren CA, Reitsma JB, Moons KG, de Groot JA. Latent class models in diagnostic studies when there is no reference standard—a systematic review. Am J Epidemiol. 2014;179:423–31. 10.1093/aje/kwt286 24272278

