# Supplementary material for: Highly Pathogenic Avian Influenza A(H5N8) Clade 2.3.4.4b Virus in Dust Samples from Poultry Farms, France, 2021
Source: Emerg Infect Dis. 2022 Jul;28(7):1446–50. doi: 10.3201/eid2807.212247 (PMC9239875; doi:10.3201/eid2807.212247)
Supplement: Appendix — Additional information on detection of highly pathogenic avian influenza A(H5N8) clade 2.3.4.4b virus in dust samples from poultry farms, France, 2021. [file 21-2247-Techapp-s1.pdf]

Article DOI: https://doi.org/10.3201/eid2807.212247

Highly Pathogenic Avian Influenza A(H5N8) Clade 2.3.4.4b Virus in Dust Samples from Poultry Farms, France, 2021

Appendix

Methods

Sampling on Farms

Tracheal swab samples were taken and tested for avian influenza virus (AIV) by using official M/H5 PCR kits and procedures. H5 PCR-positive samples were confirmed as highly pathogenic avian influenza (HPAI) A(H5N8) clade 2.3.4.4.b virus by the French National Laboratory for Avian Influenza and Newcastle Disease using the official procedures. On-farm investigations and collection and shipping of samples were performed in strict compliance with regulation and biosecurity procedures, with the authorization and supervision of official veterinary services.

Dust was collected using dry wipes, of ≈900 cm^2 (Grosseron, https://www.grosseron.com), on the building's walls and, in 51/63 farms, on feeders. The aim was to collect dust without feces, litter, or food residues to avoid PCR inhibition as much as possible. Therefore, for feeders, the food distributing pipes were preferred for automatic feeders, otherwise the dust was collected in the top part of feeders to avoid feces or food residues. On walls, the dust was sampled on all surfaces roughly above 60 cm high, which appears not to have any other particles except dust. Both sides of the wipes must be covered by dust. Wipes were shipped to the National Veterinary School of Toulouse (France), stored at 4°C, and processed within 48 hours.

Aerosol sampling using the dry cyclonic air sampler Coriolis Compact (Bertin Technologies, https://www.bertin-instruments.com) was done according to the manufacturer's instructions. In brief, the Coriolis Compact was calibrated at a 50 L/min air flow rate that enables

the dry collection of aerosol particles from 500 nm–10 µm in diameter. The 2-stage bioaerosol cyclone (BC) sampler, NIOSH BC 251, developed by the National Institute of Occupational Safety and Health (NIOSH; https://www.cdc.gov/niosh), was also used according to the manufacturer's instructions. Prior to utilization, the sampler, connected to an APEX (Casella, https://www.casella.com) personal sampling pump, was calibrated using a flow meter at 3.5 L/min. On the sampler, 15 mL and 1.5 mL collection tubes were installed, as well as the cassette in which a handmade 37 mm diameter polytetrafluoroethylene (PTFE) filter with 1.5 µm pore was installed.

During the collection time, both samplers were positioned roughly in the center of the barn at ≈1.5 m from the floor and at a minimal distance of 2 m from each other to avoid interference. The simultaneous use of both collection devices with respectively high (50 L/min) and low (3.5 L/min) air flow rates was shown to enable an accurate airborne virus detection and quantification (*1*). Aerosols were collected for 20 min with the Coriolis sampler, allowing aerosol collection of 1 m^3 of air, and between 25 and 60 min for the NIOSH samplers due to experimental constraints. All experimental samples were stored at 4°C before being processed.

Processing Methods

Prior to RNA extraction, tracheal swab and environmental samples (dust and aerosols) were processed. Tracheal swab samples were individually placed into single 1.5 mL centrifuge tubes containing 500 µL phosphate-buffered saline (PBS) and vigorously vortexed for 10–15 s. From the 20 swabs, we created 4 pools of 5 swabs by using 100 µL of each sample. Dust samples from the wipes were processed by using 20 mL PBS directly in the transport bag. After mixing by hand massage for 2–3 min, the dust solution was collected and aliquoted into 1.5 mL centrifuge tubes.

Aerosols were resuspended by using a PBS-0.5% BSA (w/v) solution; 1 mL of the solution was added into the Coriolis Compact collection cone and the first 2 stages of the NIOSH BC 251 collection tubes (15 mL falcon tubes and 1.5 mL microtubes). All samples were vigorously vortexed for 10–15 s before being aliquoted into 1.5 mL centrifuge tubes. The NIOSH BC 251 fraction 3 membrane filter was carefully collected from the cassette by using sterile pliers and placed into a 50 mL falcon. The filter was vortexed for 10 s while dry before adding 1.5 mL of the PBS-0.5% BSA solution and submitted through another vigorous vortex for

10–15 s. Samples were aliquoted into 1.5 mL centrifuge tubes. All samples were stored at −80°C after processing.

RNA Extraction and PCR Methods

RNA samples were extracted by using the magnetic bead-based ID Gene Mag Fast Extraction Kit (IDvet, https://www.id-vet.com) associated with the IDEAL 32 extraction robot (IDvet), following the manufacturer's instructions. The presence of AIV RNA was investigated by performing a 1-step, real-time reverse transcription quantitative PCR (rRT-qPCR), Influenza A Duplex kit (IDvet), targeting the matrix (M) gene and then targeting the H5 subtype from positive results by using the Influenza H5/H7 Triplex kit (IDvet).

Virus Isolation

Positive H5 subtype rRT-qPCR biologic and environmental samples from 5 different animal houses (A, B, C, D, E) were selected, based on their cycle threshold (C_t) values and global study representativity, to test for virus viability using specific virus free (SPF) embryonated chicken eggs purchased from INRAE PFIE (https://www.nadir-project.eu). SPF eggs were incubated for 9–11 days at 37°C. Infection was executed in triplicate to optimize data analysis. Different inoculum concentrations were used depending on the sampling method. Inoculum from dust sampling (walls and feeders) were diluted at 0.1 and aerosols from the NIOSH BC 251 sampler were diluted at 0.5. Inoculum from the Coriolis Compact was used at a concentration of 0.5 and a single pool of tracheal swab samples was used at a concentration of 0.01 for a single egg, and a concentration of 0.1 for the last 2 remaining eggs. All dilutions were achieved by using a sterile 1× PBS solution with penicillin (1,000 U/mL) and streptomycin (1 mg/mL). Eggs were inoculated with 150 µL of the correspondent dilution, kept in a humidity-chamber at 37°C for 48 h then at 4°C for 12 h. Allantoic fluid was collected from each egg and a hemagglutination titration was directly performed in a 96-well U-bottom plate; 100 µL of allantoic fluid was pipetted in the first plate row then the next 7 rows were filled with 50 µL PBS. A cascade of 0.5 dilutions was performed and 50 µL of 1% solution of fresh and washed chicken red blood cells were added to each well before a 25 min room temperature incubation. All samples were controlled by H5 AIV subtype rRT-qPCR by using the ID Gene Influenza H5/H7 Triplex kit (IDvet).

Samples with inconclusive results were tested twice on eggs. Hemagglutination assay was performed and HA-positive allantoic fluids were tested by rRT-qPCR for the H5 subtype to assess the presence of viral RNA.

Latent-Class Modeling

The analytical approach that was used modeled the cross-detection of farms whose true epidemiologic status (presence or absence of HPAI virus) was assessed using 4 different imperfect observation processes (based on 4 different sample types) of unknown sensitivity (defined as the probability of detecting the virus if it is present in the farm) and specificity (defined as the probability of not detecting the virus if it is absent from the farm). For each group of farms (clinically affected or not), the observed frequency of the $2^4 = 16$ different combinations of test results was assumed to have been distributed according to a multinomial distribution of parameters, n = 16 clinically affected farms (48 non-clinically affected farms) and 9 probabilities expressed as a combination of the proportion of infected farms and the sensitivity and specificity of each of the 4 sensitivity and specificity parameters. The analyses were performed in a Bayesian framework by using WinBUGS software (*2*) embedded in R software (*3*) by the R2WinBUGS library (*4*). For all sensitivity parameters, we assumed Uniform (0–1) as prior distributions. Because rRT-qPCR testing was considered to be highly specific (i.e., uncontaminated samples are very likely to test negative), Sp_{i_sample} parameters were assigned a β prior distribution defined such that its 5th percentile was equal to 90%, and its median to 98%. Given the high level of suspicion in clinically-affected flocks, we assumed a Beta (10–1) as a prior distribution for the proportion of infected flocks among clinically affected flocks. For the non-clinically affected flocks, we assumed a Uniform (0–1) prior distribution for the proportion of infected flocks. We ran 2 simulation chains of 100,000 iterations whose convergence and mixing were assessed by checking the trace plots for all monitored parameters and calculating the Gelman-Rubin convergence statistics (*5*). The first 5,000 iterations were discarded to allow for burn-in of the chains and the chains were thinned, taking every 100th sample to reduce autocorrelation among the samples.

Statistical Analysis

Differences in C_t distribution based on the sampling strategy were investigated by using a pairwise Wilcoxon test. For the calculation, negative C_t were associated to a C_t value of 40 and the mean C_t from wipes and swabs was calculated for each poultry house.

References:

1. Raynor PC, Adesina A, Aboubakr HA, Yang M, Torremorell M, Goyal SM. Comparison of samplers collecting airborne influenza viruses: 1. primarily impingers and cyclones. PLoS One. 2021;16:e0244977. PubMed https://doi.org/10.1371/journal.pone.0244977

2. Lunn DJ, Thomas A, Best N, Spiegelhalter D. WinBUGS—a Bayesian modelling framework: concepts, structure, and extensibility. Stat Comput. 2000;10:325–37. https://doi.org/10.1023/A:1008929526011

3. R Core Team. R: A language and environment for statistical computing. Vienna, Austria: R Foundation for Statistical Computing; 2014.

4. Sturtz S, Ligges U, Gelman A. R2WinBUGS: a package for running WinBUGS from R. J Stat Softw. 2005;12:1–16.

5. Gelman A, Carlin JB, Stern HS, Rubin DB. Bayesian data analysis. CRC Press: Boca Raton, Florida; 2004.

Appendix Table 1. Cycle threshold values for tracheal swab and environmental samples collected on 63 poultry houses and tested for highly pathogenic avian influenza A(H5N8), France, December 2020–April 2021*

									Aerosol sampling†			
ID		Clinical	Wipes		Tracheal swab sample pools					NIOSH BC 251‡		
no.	Species	signs	Walls	Feeder	1	2	3	4	Coriolis	1	2	3
1	MD	Y	28.3	25.2	23.5	19.5	25.2	22.4	ND	ND	ND	ND
2	G	Y	29.29	27.91	25.2	26.7	26.3	27.1	ND	ND	ND	ND
3	MD	N	28.51	–	23.85	26.92	26.12	29.49	ND	ND	ND	ND
4	C	Y	29.44	29.17	25.26	25.51	23.31	24.96	ND	ND	ND	ND
5	MD	Y	26.81	23.67	19.76	24.98	25.30	24.93	23.39	26.82	30.56	33.76
6	MD	N	31.19	27.85	19.15	22.17	17.69	22.74	ND	ND	ND	ND
7	MD	N	25.1	23.71	20.44	21.86	20.82	21.13	ND	ND	ND	ND
8	MD	N	–	–	–	–	–	–	ND	ND	ND	ND
9	MD	N	–	–	–	–	–	–	34	36.06	–	–
10	C	Y	36.27	ND	–	24.78	24.51	20.02	32.67	35.81	36.22	–
11	MD	N	27.47	25.84	25.22	24.47	22.98	27.78	31.99	34	–	–
12	MD	Y	27.53	27.02	22.99	25.95	19.84	20.08	31.63	32.44	–	–
13	Q	N	–	–	–	–	–	–	–	–	–	–
14	Q	Y	33.77	34.63	30.15	29.71	24.7	27.22	30.18	36.11	–	–
15	MusD	N	25.2	23.53	24.14	24.69	24.88	29.34	ND	ND	ND	ND
16	MusD	N	25.35	24.91	34.19	27.65	32.82	33.29	ND	ND	ND	ND
17	MD	N	25.83	–	–	–	–	–	ND	ND	ND	ND
18	MD	N	–	–	–	–	–	–	ND	ND	ND	ND
19	C	N	–	–	–	–	–	–	–	–	–	–
20	C	Y	30.19	27.32	24.55	20.18	19.53	18.69	27.92	33.66	–	–
21	MD	N	34.04	28.69	26.48	21.76	25.76	21.03	ND	ND	ND	ND
22	PD	N	32.78	31.07	–	–	–	–	ND	ND	ND	ND
23	MD	Y	28.24	25.81	22.86	20.86	22.07	22.05	ND	ND	ND	ND
24	C	N	–	33.73	–	–	–	–	ND	ND	ND	ND
25	PD	N	32.05	31.15	–	36.11	–	–	31.56	–	–	–
26	MD	N	25.48	25.08	20.7	27.85	25.54	24.4	30.54	33.64	36	35.99
27	MD	N	26.44	ND	22.56	23.83	24.1	22.56	ND	ND	ND	ND
28	MD	Y	24.55	27.28	29.83	28.11	25.8	–	ND	ND	ND	ND
29	MD	N	30.14	27.43	21.92	19.02	19.58	20.96	31.23	27.84	32.36	36.3
30	MD	Y	28.35	29.51	18.86	23.15	17.87	27.02	24.85	25.81	33.13	–
31	PD	N	30.9	33.76	–	36.13	36.02	35.95	33	–	–	–
32	PD	N	–	–	–	–	–	–	ND	ND	ND	ND
33	MD	N	25.03	22.32	22.95	25.11	22.85	25.02	ND	ND	ND	ND
34	MD	N	22.99	24.23	19.94	21.22	18.09	20.63	26.9	23.75	18.63	–

ID no.	Species	Clinical signs	Wipes: Walls	Wipes: Feeder	Tracheal swab sample pools: 1	2	3	4	Aerosol sampling†: Coriolis	NIOSH BC 251‡: 1	2	3
35	MD	Y	20.73	19.74	25.64	24.87	29.91	23.83	23.26	24.08	29.49	–
36	MD	Y	20.73	19.74	26.29	23.16	28.06	25.06	ND	ND	ND	ND
37	MD	N	31.97	30.28	27.74	–	24.46	30.03	27.52	33.01	–	36.21
38	MD	N	26.69	24.6	25.05	19.84	23.97	24.01	ND	ND	ND	ND
39	MD	N	–	27.07	21.09	19.22	22.68	19.68	ND	ND	ND	ND
40	MD	Y	24.94	24.46	21.9	24.1	29.7	21.2	ND	ND	ND	ND
41	MD	N	–	–	–	–	–	–	33.08	–	–	–
42	MD	N	–	30.77	–	–	–	–	ND	ND	ND	ND
43	MD	Y	27.59	23.99	21.71	21.98	22.65	24.72	22.15	25.55	–	35.87
44	MD	N	–	–	–	–	–	–	ND	ND	ND	ND
45	MD	N	–	–	–	–	–	–	ND	ND	ND	ND
46	MD	N	–	–	–	–	–	–	ND	ND	ND	ND
47	MD	N	–	–	–	–	–	–	ND	ND	ND	ND
48	MD	N	–	–	–	–	–	–	ND	ND	ND	ND
49	PD	N	32.3	ND	32.14	33.02	35.53	–	ND	ND	ND	ND
50	PD	N	30.8	27.3	23.9	25.3	29	29.7	ND	ND	ND	ND
51	PD	N	–	32	–	–	–	–	ND	ND	ND	ND
52	PD	N	29	21	26.2	22.3	22.3	31.1	ND	ND	ND	ND
53	PD	N	29.5	29.3	34.1	33.7	34.2	–	ND	ND	ND	ND
54	C	N	–	ND	–	–	–	–	ND	ND	ND	ND
55	C	N	–	–	–	–	–	–	ND	ND	ND	ND
56	C	N	–	ND	–	–	–	–	ND	ND	ND	ND
57	C	N	–	ND	–	–	–	–	ND	ND	ND	ND
58	C	N	–	ND	–	–	–	–	ND	ND	ND	ND
59	C	N	–	ND	–	–	–	–	ND	ND	ND	ND
60	C	N	–	ND	–	–	–	–	ND	ND	ND	ND
61	MD	N	–	ND	–	–	–	–	ND	ND	ND	ND
62	MD	N	–	ND	–	–	–	–	ND	ND	ND	ND
63	MD	N	–	ND	–	–	–	–	ND	ND	ND	ND

*C, chicken; C_t, cycle threshold; G, goose; ID, identification; MD, mule duck (a hybrid Muscovy/Peking); MusD, Muscovy duck; PD, Peking duck, Q, quail; ND, not done; NIOSH, National Institute for Occupational Safety and Health; –, no C_t value detected.
†Aerosol sampling was performed on 19 farms. Coriolis Compact (Bertin Instruments, https://www.bertin-instruments.com) and NIOSH BC 251 (National Institute for Occupational Safety and Health, https://www.cdc.gov/niosh) instruments were used.
‡NIOSH BC 251 sampling device has 3 fractions for different particle sizes; fraction 1 for >4 µm, 2 for 1–4 µm, and 3 for <1 µm.

Appendix Table 2. Official notification status of the animal houses included in a study for detection of highly pathogenic avian influenza A(H5N8) in dust from poultry farms, France, December 2020–April 2021*

Animal houses	Notification	House no.
HPAI-positive animal houses	Officially notified as outbreak	1, 2, 4, 5, 7, 10, 14, 20, 21, 23, 24, 26, 27, 28, 30, 31, 33, 34, 35, 36, 40, 43
	Official detection negative	NA
	Official detection not done	NA
Suspected animal houses adjacent to HPAI-positive poultry house	Officially notified as outbreak	49, 50, 51, 52, 53
	Official detection negative	19, 31, 41, 42
	Official detection not done	3, 6, 13, 15, 16, 22, 25, 29, 37, 38, 39, 44, 45, 46
Suspected animal houses epidemiologically related to HPAI-positive poultry house	Officially notified as outbreak	9, 11, 12
	Official detection negative	8, 47, 48, 54, 55, 56, 57, 58, 59, 60, 61, 62, 63
	Official detection not done	17, 18

*HPAI, highly pathogenic avian influenza; NA, not applicable.

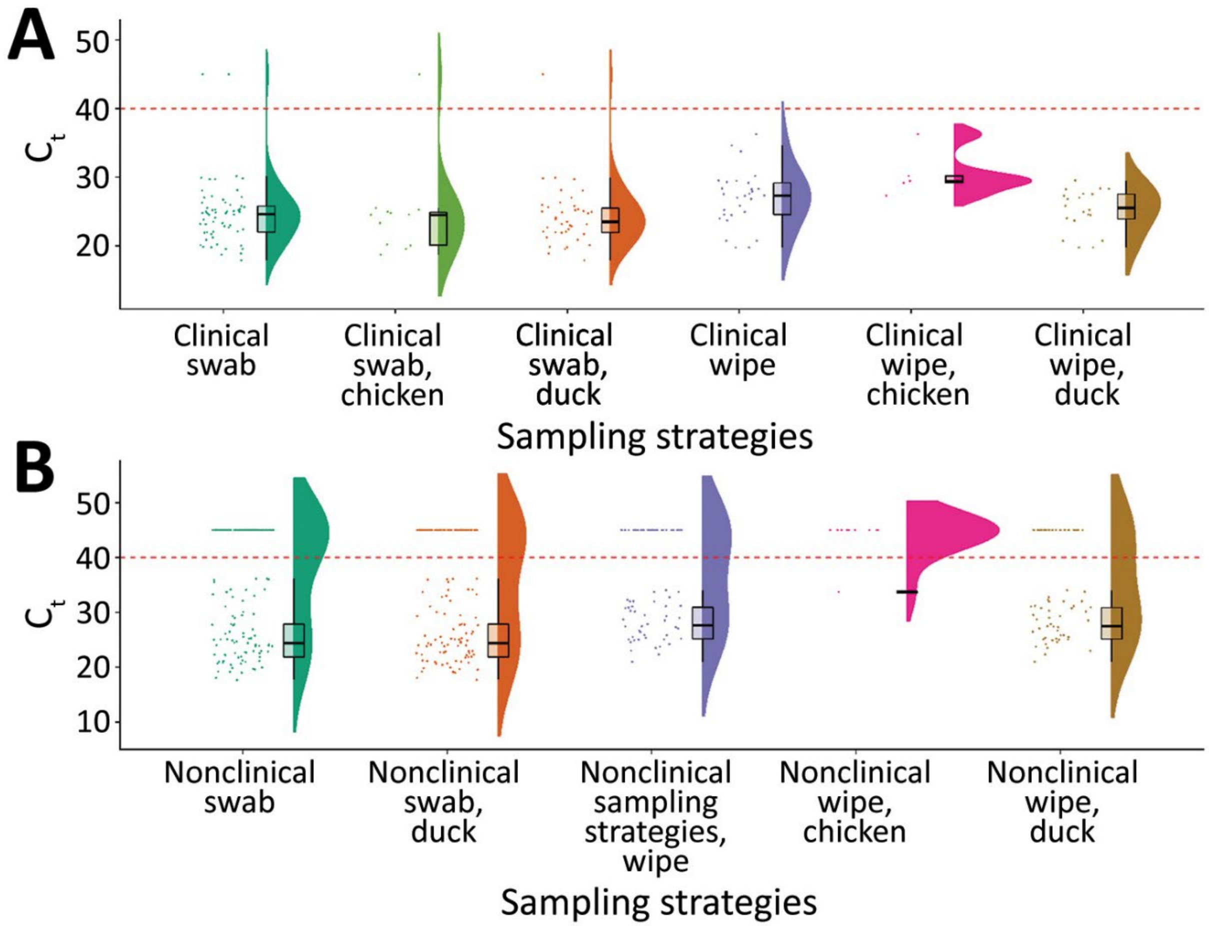

Appendix Figure. Half-violin, scatter, and box plots for C_t values of highly pathogenic avian influenza A(H5N8) virus detected from tracheal swab and environmental samples collected on poultry farms, France, December 2020–April 2021. C_t values are from results of real-time reverse transcription quantitative PCR on samples. A) Samples from 10 duck and 3 chicken houses where the animals had clinical signs of HPAI; and global results of 15 C_t values provide distribution comparison. B) Samples from 38 duck and 9 chicken houses where animals did not have clinical signs of HPAI; and global results of 48 C_t values provide distribution. Each dot indicates a C_t value from 1 wipe sample or 1 pool of 5 tracheal swab samples. Half-violins show distribution of C_t values for each sample type. Boxes show 95% CI for C_t values; horizontal lines in boxes indicate mean, vertical lines from boxes SD. Red dashed horizontal lines indicate C_t 40, the cutoff value for negative results. A pairwise Wilcoxon statistical test was used on the mean C_t values for wipes and swabs for each sampling strategy and animal house status. No statistically significant differences were found.
